# Interplay between Autophagy, Exosomes and HIV-1 Associated Neurological Disorders: New Insights for Diagnosis and Therapeutic Applications

**DOI:** 10.3390/v9070176

**Published:** 2017-07-06

**Authors:** Chet Raj Ojha, Jessica Lapierre, Myosotys Rodriguez, Seth M. Dever, Mohammad Asad Zadeh, Catherine DeMarino, Michelle L. Pleet, Fatah Kashanchi, Nazira El-Hage

**Affiliations:** 1 Department of Immunology, Herbert Wertheim College of Medicine, Florida International University, Miami, FL 33199, USA; cojha001@fiu.edu (C.R.O.); jlapi008@fiu.edu (J.L.); myrodrig@fiu.edu (M.R.); Seth.Dever@dgs.virginia.gov (S.M.D.); 2 Laboratory of Molecular Virology, School of Systems Biology, George Mason University, Manassas, VA 20110, USA; mohammad.asadzadeh@gmail.com (M.A.Z.); demarin@gmu.edu (C.D.); mlpleet@gmail.com (M.L.P.); fkashanc@gmu.edu (F.K.)

**Keywords:** autophagy, exosomes, HIV, neurodegenerative disorders, therapeutic interventions

## Abstract

The autophagy–lysosomal pathway mediates a degradative process critical in the maintenance of cellular homeostasis as well as the preservation of proper organelle function by selective removal of damaged proteins and organelles. In some situations, cells remove unwanted or damaged proteins and RNAs through the release to the extracellular environment of exosomes. Since exosomes can be transferred from one cell to another, secretion of unwanted material to the extracellular environment in exosomes may have an impact, which can be beneficial or detrimental, in neighboring cells. Exosome secretion is under the influence of the autophagic system, and stimulation of autophagy can inhibit exosomal release and vice versa. Neurons are particularly vulnerable to degeneration, especially as the brain ages, and studies indicate that imbalances in genes regulating autophagy are a common feature of many neurodegenerative diseases. Cognitive and motor disease associated with severe dementia and neuronal damage is well-documented in the brains of HIV-infected individuals. Neurodegeneration seen in the brain in HIV-1 infection is associated with dysregulation of neuronal autophagy. In this paradigm, we herein provide an overview on the role of autophagy in HIV-associated neurodegenerative disease, focusing particularly on the effect of autophagy modulation on exosomal release of HIV particles and how this interplay impacts HIV infection in the brain. Specific autophagy–regulating agents are being considered for therapeutic treatment and prevention of a broad range of human diseases. Various therapeutic strategies for modulating specific stages of autophagy and the current state of drug development for this purpose are also evaluated.

## 1. Introduction

Human immunodeficiency virus type 1 (HIV-1) enters the central nervous system (CNS) soon after infection and about half of all adults with acquired immunodeficiency syndrome (AIDS) suffer from neurological complications related to HIV-1 [1,2]. Despite a dramatic reduction in HIV-1 RNA levels and increased survival of people living with HIV-1 by long-term combination anti-retroviral therapy (cART), the virus persists in latently infected cells or viral reservoirs which can be reactivated upon discontinuation of cART [3]. In the brain, long-lived cells such as macrophages/microglia and, to a lesser extent, astrocytes are the major cell types infected with HIV-1 and potentially serve as reservoirs and sources of chronic infection [4,5]. A broad spectrum of neurological impairment including asymptomatic neurocognitive impairment (ANI), mild neurocognitive disorder (MND), and HIV-1 associated dementia (HAD) is defined within the term HIV associated neurological disorder (HAND) [6]. Though the prevalence of the severe form of HAND has decreased in the post cART era, the overall prevalence of HAND still remains high with milder manifestations [7,8]. cART can impede HIV-1 disease progression and increase the life expectancy of HIV-1 infected people, but complete cure of HIV-1 is still not available [9]. Although the exact etiology of HIV-related neurocognitive disorders in the cART era is still unknown, chronic and persistent inflammation in CNS is a common feature of HIV infection that may lead to accumulation of neurological damage [10]. 

Poor penetration of the blood brain barrier (BBB) and potential neurotoxicity by many common antiretrovirals make the eradication of viral reservoirs in the brain challenging [11,12,13,14]. Furthermore, a high mutation rate of the viral genome and its ability to undergo latency for extended periods by integrating into the host genome make curing HIV-1 a daunting task. Considering the limitations of current therapeutic approaches, many alternative methods to develop anti-HIV drugs are under investigation. Some of these approaches include gene therapy, gene editing, RNA interference, and modulation of different cellular physiological processes [15,16,17]. Modulation of the autophagy pathway has emerged as an alternative approach to decrease HIV-1 induced inflammation or HIV-1 viral load in brain [18,19,20,21]. Autophagy is a highly conserved lysosomal degradation pathway and is crucial for controlling intracellular pathogens such as viruses [22]. Autophagy is even more important for post-mitotic cells such as neurons, where abnormal cellular or viral proteins cannot be diluted by cell division (leading to their accumulation within a cell) and which requires reciprocal transport of these proteins over long distances from the cell body to dendrites or axons [20,23,24]. Owing to these facts, many researchers have been involved in evaluating the impact of modulating cellular autophagy as an adjuvant therapy for HIV-1, particularly to hinder neurological disorders and eradicate brain reservoirs. In this review, we explore autophagy and its regulation by host and viral proteins, interaction with HIV-1 infection and exosomal biogenesis, as well as discuss the current progress towards the therapeutic modulation of autophagy.

## 2. Autophagy and Its Regulation

Autophagy, the process of ‘self-eating’, was first noted by Sam L. Clark Jr. in newborn mice kidney cells, and coined by Christian de Duve who observed the degradation of mitochondria and other intra-cellular structures within lysosomes of rat livers perfused with glucagon [25,26]. Autophagy is induced by nutrient deprivation or stress and is the major cellular machinery for the clearance or recycling of long-lived proteins and organelles such as the endoplasmic reticulum, mitochondria, peroxisomes, and even the nucleus and ribosomes [26]. Autophagy promotes the energy conservation, recycling, and salvage of cellular nutrients, thereby enabling cell survival during starvation. Autophagy is also a key mechanism for protein homeostasis and quality control [27]. Basal levels of autophagy are crucial not only for removal of long-lived proteins and superfluous or damaged organelles, but also for cellular development and differentiation [28], innate and adaptive immunity [29,30], and programmed cell death (type II) [31,32]. 

More than 30 autophagy related (ATG) proteins are involved in the complex processes of autophagosome formation, encapsulation of target cargoes and subsequent fusion with the lysosome for degradation (reviewed in [33,34]). Autophagosome formation involves initiation, nucleation, and expansion of the isolation membrane [27]. Initiation begins with the formation of the phagophore assembly site (PAS), the origin of which is still unclear in mammals [26,35]. The UNC51-like kinase (ULK) complex consisting of ULK1 (or ULK2), autophagy-related protein 13 (ATG13), focal adhesion kinase (FAK) family kinase interacting protein (FIP200) and ATG101 assembles to the PAS [35]. Subsequent nucleation requires a class III phosphoinositide 3 kinase (PI3K) complex composed of the vacuolar protein sorting 34 (VPS34) along with its regulatory subunits ATG14L, VPS15 and Beclin-1 (ATG6 homologue), for the production of a pool of phosphatidylinositol 3-phosphate that is specific to autophagosomes. Following nucleation, recruitment of ATG5-ATG12-ATG16L to the autophagosome membrane facilitates the conjugation of phosphatidylethanolamine (PE) to LC3 (microtubule-associated protein 1 light chain 3). Lipidated or PE-conjugated LC3 (LC3–PE) is required for the expansion of autophagosome membranes, recognition of target cargo, and fusion of the autophagosome with lysosomes. The autophagosome then fuses with endocytic and lysosomal compartments, ultimately leading to formation of autolysosome [27,34].

Many signaling molecules induced by cellular and environmental stimuli are involved in the regulation of autophagy. Among them, mammalian target of rapamycin complex 1 (mTORC1) is the most well characterized and important negative regulator of autophagy. mTORC1, stimulated by the presence of amino acids, ATP, or growth factors, inactivates ATG-1 (ULK1) so that the initiation complex cannot be formed. Under nutrient starved or amino acid deprived conditions, mTORC1 is inactivated, allowing for the progression of autophagy [36]. On the other hand, AMP-activated protein kinase (AMPK) promotes autophagy by activating ULK1 and interrupting its interaction with mTORC1. AMPK itself is an energy sensor stimulated by starvation [37]. It also phosphorylates Beclin-1 and promotes its incorporation in PI3K complex I [38]. Beclin-1 under normal conditions remains associated with other proteins, particularly Bcl-2 and sometimes 14-3-3 or vimentin 1. Dissociation of Beclin-1 from Bcl-2 is mediated either by phosphorylation of the BH3 domain of Beclin-1 by death associated protein kinase (DAPK) or through the phosphorylation of Bcl-2 by c-jun N terminal kinase (JNK) which is essential for Beclin-1 to be incorporated in the PI3K complex I [39,40]. Akt and protein kinase B (PKB) favor the interaction of Beclin-1 with vimentin 1 and 14-3-3, resulting in the inhibition of autophagy [41].

Though the exact mechanism is unknown, LC3 phosphorylated by PKA and PKC becomes refractory to autophagosome formation [42]. The adaptor protein sequestosome-1 (SQSTM1) (p62) is also negatively regulated by phosphorylation. Casein kinase 2 (CK2) phosphorylates p62 and enhances its interaction with ubiquitinated proteins for selective clearance by autophagy [43]. Insulin/Insulin like growth factor (IGF-1), DNA damage regulated autophagy modulator (DRAM), p53, Forkhead box O (FOXO) and reactive oxygen species (ROS) are some of the other factors that regulate autophagy at various points (reviewed in [34]). Autophagy is also regulated by epigenetic modification, most importantly arginine methylation (H3R17me2) mediated by coactivator associated arginine methyltransferase 1 (CARM1) during starvation [44]. Inhibition of CARM1 or H3R17me2 greatly impairs starvation-induced autophagy [44].

## 3. Autophagy and Exosomes

As previously mentioned, maturation of PAS into the autophagosome, followed by fusion of the autophagosome and lysosome results in formation of the autolysosome where cellular contents are degraded [19]. Perpendicular to this process lies the endosomal pathway. Several cellular processes including endocytosis and macropinocytosis result in the formation of an endosome. Endosomes can mature into multivesicular bodies (MVBs) through inward budding of their membrane to create intraluminal vesicles (ILVs) [45]. Once formed, MVBs can go through two separate courses: (a) the MVB (or the early endosome) can fuse either directly with a lysosome, or alternatively it can fuse with the autophagosome to become an amphisome prior to fusion with a lysosome, ultimately leading to content degradation; or (b) the MVB can fuse with the plasma membrane to release its intraluminal vesicles as exosomes into the extracellular space [31,46]. Exosomes are small, nano-sized vesicles released from virtually all cell types. They play roles not only in the removal of cellular components, but also as intercellular messengers through the delivery of proteins, genomic material, and RNAs including miRNAs to neighboring cells (reviewed in [47,48]). That these two endosomal and autophagic processes are linked means that there are consequences for the biogenesis of exosomes depending on the cellular condition driving or inhibiting autophagy. A blockage of autophagosome maturation or fusion with a lysosome would result in the buildup of cellular waste in the form of a double membraned vesicle. It is then plausible that the cell would seek to rid itself of its waste through the export of autophagosomal contents outside the cell, perhaps through the fusion of autophagosomes to MVBs and release in exosomes. Alternatively, under cellular starvation conditions, the equilibrium would be shifted towards greater autophagic degradation, and a reduced biogenesis of exosomes. As such, induction of the autophagy pathway was shown to decrease in exosomal release by promoting fusion of MVBs with autophagosomes, while impaired autophagy may lead to increased exosome secretion [49,50]. This dynamic interaction between these linked pathways then may be of great significance in the context of cellular stress, such as viral infection.

## 4. Viral Regulation of Autophagy

Autophagy in viral infections has been shown to be either pro- or anti-viral, depending on the cell type, cellular environment, and the virus in question. In both cases, several viruses have evolved mechanisms to subvert the autophagic pathway for their benefit. For those situations in which autophagy has been shown to be anti-viral, several viruses have developed methods to inhibit various stages of autophagosome maturation or autolysosome formation [51]. For example, Herpes simplex virus type 1 (HSV-1) encodes two proteins, US11 and ICP34.5, that can inhibit two separate stages of the autophagy pathway [52,53]. US11 directly inhibits protein kinase RNA-activated (PKR) and thereby inhibits the signaling to initiate autophagy [52]. ICP34.5, meanwhile, can prevent autophagosomal formation by binding to Beclin-1 [53]. This tactic of Beclin-1 inhibition is shared by other viral proteins, including orf16 of Kaposi Sarcoma-associated Herpes virus (KSHV) and M11 of Murine Gamma herpesvirus 68 (MHV-68), which are homologs of Bcl-2 [54,55]. The influenza A matrix-2 (M2) protein inhibits amphisome formation (fusion of autophagosome with endosome), which is dependent on its binding to LC3 [51,56,57]. Likewise, the P protein encoded by Human Parainfluenza Virus Type 3 (HPIV3) binds to soluble N-ethylmaleimide sensitive fusion protein (NSF) attachment protein 29 (SNAP29), an intermediate SNAP receptor (SNARE) protein, thereby inhibiting fusion of the autophagosome with an endosome or lysosome [58]. 

In contrast, many other viruses induce autophagy pathway which could be beneficial for their survival and replication inside the cells. Poliovirus proteins 2BC and 3A, when co-expressed, stimulate an autophagic response. 3A will cause swelling of the endoplasmic reticulum (ER), while 2BC will result in LC3 modification. When present together inside host cells, double-membraned vesicle formation will take place [51,59,60]. Other viral proteins that stimulate autophagic signaling include the T antigen of Simian Virus 40 (SV40), Rta transcription factor of Epstein-Barr virus (EBV), the X protein of Hepatitis B virus (HBV), and three proteins (NS4B, NS5A, and NS5B) from Hepatitis C virus (HCV) [51,61,62,63,64,65,66,67,68,69]. Active cellular autophagy appears to aid in replication of other viruses such as Chikungunya and encephalomyocarditis viruses [70,71]. Interestingly, some viruses find it favorable to induce the signaling towards initiation of autophagosome formation, but also encode proteins to prevent its maturation or degradation. Some examples include influenza A, EBV, HPIV3, and Coxsackievirus B3 (CVB3) [51,63,72].

## 5. Complex Interaction between HIV-1 and Autophagy

### 5.1. HIV-1 Induces Autophagy

**Figure 1:**
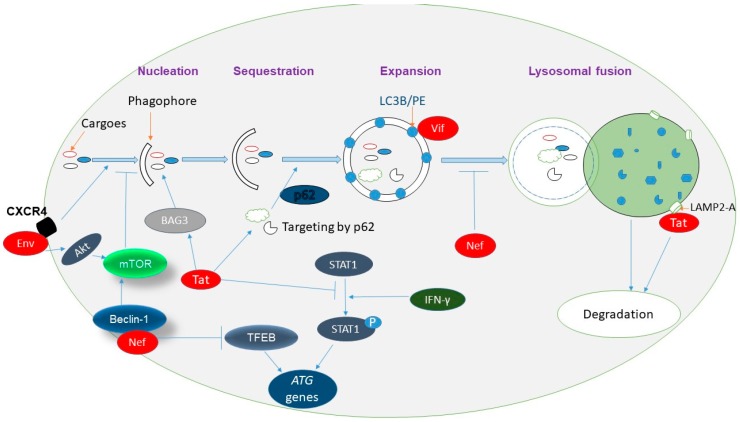
Interaction of different human immunodeficiency virus type 1 (HIV-1) proteins with the autophagy pathway. HIV-1 transcription transactivator (Tat) stimulates autophagy by increasing Bcl-2 associated athanogene 3 (BAG3) levels in human glial cells. On the other hand, Tat interferes with interferon (IFN)-γ-induced autophagy through the suppression of signal transducer and activation of transcription 1 (STAT1) phosphorylation, resulting in the reduced expression of autophagy genes including microtubule-associated protein 1 light chain 3 (LC3B). Interaction of Tat with sequestosome 1 (SQSTM1) leads to the targeting of Tat to lysosomal-mediated degradation via selective autophagy. Tat also interacts with lysosomal associated membrane protein 2A (LAMP2A), suggesting its role in lysosomal fusion in neurons. HIV-1 negative expression factor (Nef) is an anti-autophagic maturation factor which interacts with Beclin-1 resulting in activation of mammalian target of rapamycin (mTOR) and phosphorylation and cytosolic sequestration of transcription factor EB (TFEB), decreasing the biosynthesis of ATG proteins. HIV-1 envelope (Env) induces autophagy after binding to C-X-C chemokine receptor type 4 (CXCR4) on bystander CD4^+^ T cells. HIV viral infectivity factor (Vif) interacts with LC3B on the surface of autophagosomes and can block autophagic flux.

The interaction between HIV-1 and autophagy is complex and varies depending on the cell type and infectious state [73,74,75]. HIV-1 infection has been shown to induce autophagosome formation and increase protein expression of Beclin-1 and LC3 in Jurkat cells and CD4^+^ T cells [76]. Espert et al. have reported that HIV-1 envelope (Env) glycoproteins trigger autophagy after binding to C-X-C chemokine receptor type 4 (CXCR4) on bystander CD4^+^ T cells, as evidenced by rapid accumulation of Beclin-1 followed by apoptosis (Figure 1) [77]. This mechanism is most likely a contributing factor to the immunodeficiency caused by CD4^+^ T cell depletion. Another study has shown that gp41 fusion activity was responsible for Env-mediated autophagy [78]. The exact role of Env-induced autophagy is still unclear; autophagy may be triggered to rescue the CD4^+^ T cells from cell death or, alternatively, to kill uninfected CD4^+^ T lymphocytes. It has been hypothesized that a reduction in cell mass by autophagy before apoptosis may facilitate the phagocytosis of infected cells [77]. HIV-1 transcription transactivator (Tat) protein, one of the HIV-1 virulence factors, is also able to stimulate autophagy by increasing Bcl-2 associated athanogene 3 (BAG3) levels in human glial cells [79]. Similarly, the interaction of Tat with lysosomal associated membrane protein 2A (LAMP2A) suggests that Tat induces autophagosome formation and lysosomal fusion in neurons [80]. Moreover, autophagy was found to be increased in postmortem brains from human subjects with HIV-1-associated encephalitis [18]. These findings may indicate that autophagy is induced by intact HIV-1 or one of its components, though the exact mechanism may vary depending on cell type.

### 5.2. Autophagy Restricts HIV-1 Infection and Disease Progression

Although the exact mechanism of autophagy mediated restriction against HIV-1 infection in different cells is still unclear, it is reported that cellular autophagy provides some sort of immunity against HIV-1 infection and replication [73,81]. One of the possible mechanisms might be the interaction between p62/SQSTM1 and HIV-1 Tat, resulting in the targeting of Tat to lysosomal-mediated degradation via selective autophagy, as shown by a recent study using CD4^+^ T cells (Figure 1) [81]. This interaction was independent of ubiquitination and was not mediated by conventional domains of p62, the ubiquitin associated domain (UBA) or the PB1 domain. Moreover, Tat originating from infected cells entering into uninfected neighboring cells can also be degraded by autophagy using a similar mechanism [81]. In a separate study, significantly higher levels of autophagosomes and autophagosomal markers were detected in peripheral blood mononuclear cells (PBMCs) from HIV-1 infected long term non-progressors and elite controllers in comparison to normal progressors [82]. Interestingly, HIV-1 virions were detected only in weakly autophagic macrophages, not in the macrophages with higher numbers of autophagosomes [19]. Our group has also shown that activation of the host autophagic pathway by HIV-1 infection represents an essential mechanism in controlling viral replication and virus-induced inflammatory responses in microglia [73]. These findings further support that autophagic vesicles restrict HIV-1 replication, possibly by targeting viral components for degradation.

### 5.3. HIV-1 Has Evolved Mechanisms to Defend against Autophagy

Although HIV-1 induces autophagy in the initial phase of primary infection, it has evolved various mechanisms to counteract or block autophagy by the action of several viral proteins possibly in order to avoid degradation in a cell type dependent manner [83,84,85,86]. We have reported increased levels of Beclin-1 and LC3B proteins accompanying accumulation of LC3 reporter red-green fluorescent protein (RFP-GFP) (yellow) puncta, suggesting HIV-1 mediated blockage of lysosomal degradation in microglia [73]. The C-terminus of HIV viral infectivity factor (Vif) has been shown to interact with LC3B in productively infected primary CD4^+^ T cells, resulting in the blockage of autophagosome formation (Figure 1) [83]. Another HIV-1 protein, negative expression factor (Nef) produced in actively infected macrophages, interacts with Beclin-1 resulting not only in the activation of mammalian target of rapamycin (mTOR) but also in phosphorylation and cytosolic sequestration of TFEB, which is involved in the biosynthesis of ATG proteins. Both of these consequences lead to the inhibition of autophagy, indicating that HIV-1 Nef is an anti-autophagic maturation factor [84]. Further exploration by a separate group showed that HIV-1 Nef mimicked bafilomycin A1, a fusion inhibitor, to dysregulate autophagy in human astrocytes [87]. Autophagy is blocked in dendritic cells by HIV-1-Env-mediated Akt activation, resulting in decreased viral antigen presentation to CD4^+^ T cells by major histocompatibility complex II (MHC-II) [86,88]. HIV-1 Tat has been shown to interfere with IFN-γ-induced autophagy through the suppression of STAT1 protein phosphorylation, resulting in the reduced expression of autophagy genes including LC3B. By this mechanism, HIV-1 Tat ultimately inhibits the fusion of autophagosomes with lysosomes [85].

## 6. HIV-1, Exosomes and Autophagy

HIV-1 has been shown to exploit the exosomal pathway for its own viral budding [89,90]. Several of the same proteins used for the selective packaging of cargo and budding of exosomes including proteins from the endosomal sorting complexes required for transport (ESCRT) such as TSG101 have been demonstrated to aid in the production of viable HIV-1 virions from host cells [91,92,93]. Along these lines, HIV-1 components have been likewise found in exosomes. HIV-1 proteins (Gag and Nef), RNA, and HIV-1 derived miRNA have all been reported in exosomes isolated from the blood of HIV-1 infected patients, as well as from the culture supernatant of infected cells [90,94,95,96]. The consequences of encasement of HIV-1 RNA within exosomes result in decreased levels of HIV-1 RNA available for packaging in viral particles [95]. This implies that the delivery of viral RNA to exosomes for ultimate lysosomal degradation might be a host defense mechanism to combat HIV-1 infection. Alternatively, packaging of these viral components into exosomes may have significant negative impacts on the host during the course of HIV-1 pathogenesis [97]. Exosomes containing trans-activation response element (TAR) RNA from HIV-1 infected cells have been shown to prime naïve recipient cells for infection, allowing for more efficient spread of the virus [98]. Exosomes containing the Nef protein have been shown to induce apoptosis in bystander CD4^+^ T-cells, with considerable implications for in vivo pathogenesis [99]. TAR RNA within exosomes has been shown to induce the prolific production of pro-inflammatory cytokines from primary macrophages, potentially stimulated through the activation of Toll like receptors (TLRs) and downstream NF-κB by TAR [100]. HIV-1 infection has additionally been demonstrated to result in an increase in the exosomal marker CD63 on exosomes, perhaps to enhance the uptake of exosomes from infected cells into naïve recipient cells [98,100]. These data may suggest that HIV-1 may influence the biogenesis of exosomes to better propagate infection within an infected individual. Though exosomes have been shown to play a critical role in HIV-1 infection and pathogenesis, it is still controversial whether these effects are pro- or anti-viral. Therefore, more research on exosomes in HIV-1 infection is needed. As previously mentioned, exosome formation and release is an alternative pathway to alleviate cellular stress and maintain cellular homeostasis in the case of compromised autophagy. Therefore, the ability of HIV-1 to inhibit autophagosomal degradation shifts the removal of cellular “waste”, including viral products, towards exosomal export from the cell rather than degradation. Along these lines, dysregulated autophagy in infected cells, including those in the CNS, increases the secretion and spread of exosomes containing viral material, which in turn contributes to HIV-1 induced bystander CD4^+^ T-cell apoptosis, viral spread, and neurodegeneration [98,99]. In this regard, the induction of autophagy during infection may inhibit the biogenesis of exosomes and the spread of viral proteins encapsulated therein [101].

## 7. Autophagy Modulation by HIV-1 in Central Nervous System Cells

### 7.1. Neurons

Neurons are highly specialized for intercellular communication with their axon, dendrites and synapses. The axon transports proteins and organelles over long distances via the axonal transport system. Neurons are post-mitotic, and therefore more vulnerable to accumulation of toxic proteins and organelles. As such, constant autophagic control of proteins and organelles like mitochondria is critical for proper neuronal function. High energy demand and protein turnover rates in synapses further emphasize the need of functional autophagy in neurons (reviewed in [24]). Studies have shown that HIV-1 Tat secreted from infected glia may affect bystander neurons [21,102]. HIV-1 Tat has been reported to cause an accumulation of abnormal autophagosomes and a decrease in p62 and LC3B in primary murine neurons and neuronal cell lines derived from rat neuroblastoma [80]. Bafilomycin A1-induced autophagy blockage was lessened by Tat, which colocalizes with autophagosome and lysosome markers in neuronal cells [80]. It is known that HIV-1 Tat induces neuroinflammation and neurodegeneration. HIV-1 Tat-induced acute neurotoxicity is enhanced by bafilomycin A1, but reversed with rapamycin co-treatment or LAMP2A overexpression [80]. Other studies have also shown that inhibition of neuronal autophagy can be mediated by the excitotoxic and inflammatory factors secreted by infected glia leading to neurodegeneration [103,104]. Our group reported differential *ATG* gene expression in post mortem brain tissues from HIV-1 infected subjects with or without neurocognitive impairment (NCI). When the effect of supernatant from HIV-1 infected microglia and HIV-1 Tat protein was compared in combination with morphine in neurons in vitro, we found that the glial supernatant and morphine caused significant reductions in autophagic flux and dendritic length, while Tat and morphine exposure resulted in lower autophagic activity at the initial phase and higher activities later. These results suggest that autophagy genes and their corresponding proteins may be differentially regulated at the transcriptional, translational, and post-translational levels in the brain during the various stages of HIV-1 infection, and that infected individuals exposed to morphine can experience mixed signaling of autophagic activity which could lead to more severe NCI than those without opioid use [21]. Therefore, substance-abusing individuals may require more aggressive and multifaceted treatment regimens targeting the direct and indirect cellular causes of neuro-complications from HIV-1 infection than non-substance users [21].

### 7.2. Microglia

Microglia, the resident immune cells of the CNS, are productively infected by HIV-1 and are the key players in the development of neurological disorders related to HIV-1 [73,105]. Besides phagocytosis, microglia are involved in surveillance of the microenvironment, communicating with neurons and other glia, and the release of soluble factors. Various endogenous and exogenous stimuli including viral infections can induce the activation of microglia, which in turn initiate immune responses. Controlled activation of microglia is critical for wound repair, microenvironment reconstruction, and clearance of pathogens, but exaggerated activation might have damaging effects. Autophagy is one of the major pathway that controls the state of microglial activation [105]. Our group has reported that HIV-1 infection induces autophagy in microglia by increasing the conversion of LC3I to LC3II and increasing expression of Beclin-1 and ATG5 proteins. However, co-exposure with HIV-1 and morphine decreased virus-induced autophagosome formation and overexpression of Beclin-1, probably through increases in intracellular pH which inhibits the formation of acidic vesicular organelles. Blocking the autophagy pathway with small interfering RNA targeting the *BECN1* gene (siBeclin-1) inhibited HIV-1 replication, indicating that autophagy is involved in mediating HIV-1 replication. However, in combination with morphine, viral replication, but not morphine and viral-induced inflammatory responses, was enhanced in siBeclin1-treated cells, suggesting a Beclin-1 independent mechanism in the interaction of HIV-1 replication and morphine in microglial cells [73].

### 7.3. Astrocytes

In the mammalian brain, astrocytes are the most abundant cell type and are responsible for the maintenance of brain homeostasis. They are involved in many crucial functions in the brain such as neurotransmitter trafficking and recycling, nutrient and ion metabolism, and defense against oxidative stress [106]. HIV-1 can infect astrocytes which serve as viral reservoirs or maintain low levels of viral replication [107,108]. It has been shown that HIV-1 suppresses autophagy, probably in the later stage of infection, inducing astrocyte toxicity. Autophagic stimulation therefore protects neuro-glial cells from this HIV-1-induced toxicity [109]. Moreover, the HIV-1 protein Nef, which is expressed abundantly in infected human astrocytes, blocks autophagic degradation that may lead to neuropathogenesis [87]. A more recent report showed that the HIV-1 protein gp120 in combination with methamphetamine additively induced autophagy in astrocytes, and inhibition of autophagy resulted in acceleration of cell death induced by methamphetamine and gp120 [110]. This suggests that autophagy functions as a protective response against apoptosis caused by methamphetamine and gp120. Furthermore, current studies conducted by our group support the role of autophagy as a key mediator in regulating brain homeostasis, HIV replication and viral and morphine-induced inflammatory cytokines in astrocytes [111].

## 8. Therapeutic Approaches for HIV-1 Based on Autophagy

The modulation of autophagy is currently being investigated for the treatment of cancer [112,113,114], inflammatory bowel disease [115], proteopathies such as Huntington’s disease [116,117], and infectious diseases such as tuberculosis [118]. Given the capacity of autophagy to work at the host cellular level to improve intracellular killing of pathogens, modulating autophagy may potentially improve the outcome of cART to treat HIV infection. Moreover, as autophagy is a cellular process, development of viral resistance is less likely.

### 8.1. Induction of Autophagy

Induction of autophagy in patients under cART may be a potential approach to boost HIV-1 treatment strategies [119]. There are a variety of approaches to induce autophagy that have been investigated for HIV-1 suppression. Inducing the autophagy pathway would be an added value to the existing treatments that target different stages of the HIV life cycle for HIV-1 infected patients.

#### 8.1.1. mTOR Inhibitors

Rapamycin, an allosteric inhibitor of mTOR (complex 1 only) and autophagy inducer, interferes with viral entry of CCR5-tropic HIV and with basal transcription of the HIV long terminal repeat (LTR), potently inhibiting replication of R5 tropic HIV in primary cells [120,121,122]. Rapamycin is a clinically approved therapeutic agent for the lung disease lymphangioleiomyomatosis and to prevent organ transplant rejection; however, it has also shown anti-HIV-1 properties in vitro as well as in vivo [123]. Furthermore, rapamycin works synergistically with viral entry inhibitors such as enfuvirtide and vicriviroc to enhance their activity [121]. The potential usefulness of rapamycin in vivo is challenged by its immunosuppressive nature and adverse effect on metabolism [124]. Recently, ATP-competitive inhibitors of mTOR (TOR-KI) have been developed which can inhibit both mTORC1 and mTORC2 complexes [125,126]. INK128, a prototype of TOR-KI, is safer than rapamycin and inhibits both CCR5 and CXCR4 tropic HIV-1 in primary lymphocytes [122,127]. It also inhibited basal as well as induced transcription of HIV-1 RNA [12]. Importantly, it synergistically enhances the antiviral potency of maraviroc (a CCR5 antagonist) and other antivirals targeting reverse transcriptase, integrase and protease [122]. INK128 decreased plasma HIV-1 RNA and partially restored CD4/CD8 cell ratios in preclinical animal models, notably humanized mice [122]. Similarly, torin-1, a highly potent selective TOR-KI, can directly inhibit both mTORC1 and mTORC2 [128], and also reduces HIV-1 production in CD4^+^ T lymphocytes [81]. Metformin, a widely used antidiabetic agent, activates AMPK which in turn enhances autophagy either by mTOR inhibition or direct ULK1 activation [129].

#### 8.1.2. Tat-Beclin-1 Fusion Peptide

A fusion peptide has been constructed by combining the transduction domain of HIV-1 Tat protein and amino acids 267–284 of Beclin-1 by a diglycine linker [119]. The fusion peptide named Tat-Beclin-1 binds to Nef protein of HIV-1 and induces autophagy by interacting with golgi-associated plant pathogenesis-related protein 1 (GAPR-1), a negative regulator of autophagy [119]. Studies have shown that the Tat-Beclin-1 peptide suppresses the replication of many viruses in vitro and reduces mortality of mice infected with some viruses such as Chikungunya, Sindbis and West Nile virus [119]. The peptide also decreases the polyglutamine expansion protein aggregates which lead to disruption of protein folding and the infectivity of the above-mentioned viruses. Dose dependent inhibition of HIV-1 replication by nontoxic doses of Tat-Beclin-1 indicates the potential therapeutic implications of this peptide in HIV patients [119].

#### 8.1.3. Vitamin D, Trehalose and Nitric Oxide Inhibitors

The association between HIV-1 disease progression and vitamin D deficiency is well characterized [130,131]. Physiological concentrations of vitamin D and its active metabolite, vitamin D3, have been shown to demonstrate anti-HIV effects by inducing autophagy by two overlapping pathways [132,133,134]. First, 1,25-D3 binds to the vitamin D receptor (VDR) to promote formation of the PI3KC3 kinase complex, which in turn leads to autophagosome elongation and lysosomal fusion. Secondly, the microbial peptide cathelicidin is upregulated following the interaction of 1,25-D3 with VDR, resulting in the fusion of autophagosomes with lysosomes [130,134,135]. There are few data available on the effect of vitamin D supplementation in HIV-1 infection. A study has shown that the combination of alendronate with calcium and vitamin D supplementation is effective for the treatment of decreased bone mineral density in HIV-1 infection [136]. In addition to proper bone mineralization, vitamin D supplementation may have potential in controlling HIV-1 replication and limiting the rate of disease progression, increasing CD4^+^ cell counts, controlling opportunistic infections, and decreasing the risk of HAND. The establishment of the optimum dosage of vitamin D supplementation is crucial for its therapeutic use in HIV-1 treatment as described in a perspective by Spector SA [135]. 

Trehalose, a natural disaccharide present in many non-mammalian species, inhibits cellular glucose import through solute carrier 2A transporters (SLC2A, also known as glucose transporter or GLUT), and stimulates autophagy through AMPK [137]. Trehalose also acts as a free radical scavenger and cryoprotective agent [137]. Similarly, trehalose, which is an mTOR independent autophagy inducer, exerts an additive effect to rapamycin on the clearance of aggregate-prone proteins in Huntington’s disease because of increased autophagic activity [138].

Nitric oxide (NO) is an important biologically active molecule and a potent cellular messenger that plays a key role in host defense against pathogens and tumor cells [139]. Overproduction of NO is reported in HIV-1 infection and may contribute to HAND [140]. NO may affect viral pathogenesis by various mechanisms such as direct antiviral effects, impairment of the innate immune response, oxidative stress and suppression of autophagy. NO can also inhibit autophagy through the disruption of hVps34/Beclin-1 complex formation and activation of mTORC1 [139]. Inhibition of nitric oxide synthase by N-L-arginine methyl ester (L-NAME) has been shown to enhance the clearance of autophagy substrates and to inhibit neurodegeneration in a Huntington’s disease model [139]. This approach may also be applied to develop therapy against HIV-1-induced neurodegeneration. However, evidence has shown that NO activity blocks HIV-1 replication by inhibiting viral enzymes including reverse transcriptase and cellular nuclear transcription factor (NF-κB) [141]. Thus, further studies are needed to rule out the possible negative effects of inhibition of NO synthesis.

#### 8.1.4. Histone Deacetylase Inhibitors

Histone deacetylases (HDACs) regulate HIV-1 latency either directly by inducing histone deacetylation at HIV-1 integration sites or indirectly through modification of non-histone proteins such as NF-κB. HDAC inhibitors (HDACi) reposition transcription factors in the vicinity of the HIV-1 proviral genome and activate the latent virus. At the same time, HDACi are able to induce autophagy [142]. Therefore, another approach of eradicating viral reservoirs may be through activating the latent virus with HDACi, followed by killing with cART and induced autophagy. Consistent with this concept, Campbell et al. have reported that HIV-1 release from macrophages was decreased by HDACi in a dose-dependent manner via degradation of intracellular HIV-1 through autophagy induction [143]. Valporic acid is the global HDACi that depletes resting CD4^+^ T cell infection. Vorinostat or suberoylanilide hydroxamic acid (SAHA), a more potent class 1 HDACi, can induce the expression of latent HIV-1 from the resting CD4^+^ T cells of HIV-infected, cART-treated, aviremic patients [144]. Many HDACi and bromodomain-containing proteins are now being pursued as therapeutic agents to enhance current antiretroviral treatment and some of them are under clinical trials [144,145]. Although this approach seems to be effective in controlling HIV-1 infection, the off-target effects are the major concern and CNS cells were less responsive to the treatment [146]. Careful consideration of the off-target effects is prerequisite before using HDACi as a component of a multipronged therapeutic approach to eliminate latently infected cells [143]. 

### 8.2. Inhibition of Autophagy

Excessive or inappropriate activation of autophagy can lead to apoptosis (programmed cell death type I) or the alternative cell death pathway (type II) [147]. The presence of increased autophagy markers in post-mortem brains of known HIV-associated dementia patients suggests that aberrant autophagy can lead to cognitive deficits [148]. Thus, inhibition of autophagy, particularly during the active phase of HIV-1 infection, may be an alternative approach for supplementary treatment of HIV-1 infection. Chloroquine and its analog hydroxychloroquine, which are anti-malarial and anti-rheumatoid agents, mediate autophagy inhibition at the later stages of the pathway specifically by mediating lysosomal dysfunction [149]. Besides inhibition of autophagy, chloroquine can directly inhibit HIV-1 replication by preventing maturation of gp120 [150]. Interestingly, maternal exposure to chloroquine in the later stage of pregnancy has been shown to reduce vertical HIV-1 transmission [151]. An additive anti-HIV-1 effect of chloroquine when combined with zidovudine and hydroxyurea was reported in an in vitro study [152]. The dual effects of chloroquine and related compounds may be employed in the therapy of HIV-1 infection. Though the use of (hydroxyl)chloroquine is limited by its high effective dose, dimeric forms of chloroquine such as Lys01 and Lys05 have a better therapeutic index [153]. Bafilomycin A1, a macrolide antibiotic and specific inhibitor of vacuolar type H (+) ATPase, inhibits acidification of the lysosome and fusion with autophagosomes, thereby blocking autophagy progression. Furthermore, bafilomycin A1 induces the binding of Beclin-1 with Bcl-2 leading to inhibition of autophagy and promotion of apoptosis. However, inhibition of autophagy by bafilomycin A1 requires a high concentration, making it inappropriate for therapeutic use [154]. Protein biosynthesis inhibitors such as cyclohexamide and acid protease inhibitors such as leupeptin also block autophagy and cause accumulation of autophagosomes. Class III PI3K inhibitors such as 3 methyl adenine (3-MA), Wortmannin and Ly294002 also suppress autophagy [153]. However, none of these compounds have been evaluated for their therapeutic implications in HIV-1 infection.

### 8.3. Silencing of Beclin-1

Because of the complex interaction with HIV-1 and autophagy, induction of autophagy will not always suppress HIV-1 replication; rather, a balanced flux of autophagy is more important than simply inducing autophagy [128]. Many RNA viruses including HIV-1 have evolved mechanisms to block autophagosome fusion with lysosomes resulting in an accumulation of autophagosomes [56,155]. The mechanism(s) leading to accumulation of autophagosomes is still not clear, but evidence indicates that these viruses target Beclin-1, and possibly stabilize its complex with Bcl-2, leading to blockade of autophagosome maturation [156]. Moreover, lysosomal dysfunctions may further block autophagic flux [157]. Therefore, silencing of Beclin-1, as shown by us in [73], might be another approach for the treatment of neurodegenerative effects mediated by HIV-induced inflammation.

### 8.4. Downregulation of ATG Genes

Four genes (*ATG7*, *ATG12*, *ATG16L2*, *MAP1LC3B*) involved in the nucleation and elongation of autophagosomes and two genes (*CLN3* and *LAPTM5*) related to lysosomal function have been found to be HIV-1 dependency factors (HDFs) by using a large scale small RNA interference screen [148]. Inhibition or silencing of these autophagy-related genes might restrict HIV-1 infection or replication. In a study, strong inhibition of HIV replication without cytotoxicity was reported in *ATG5* or *ATG16* knock down cell lines, and a double knockdown had an additive effect on inhibition of HIV replication [158].

### 8.5. Upregulation of SQSTM1/p62

Autophagy has a significant role in antigen processing and presentation [159]. An autophagy adaptor protein, sequestosome 1 (SQSTM1)/p62 selectively targets viral antigens to the autophagy pathway and bypasses proteosomal degradation. p62 binding to the C-terminal end of HIV-1 Gag p24 results in efficient delivery of p24 into autophagosomes. Presentation of p24 to CD4^+^ and CD8^+^ T cells resulted in their differentiation into virus-specific interferon γ-producing T cells in a mouse model. This strategy may be employed to develop T-cell-based vaccines in humans [160].

### 8.6. Targeting Viral Proteins That Directly Affect Autophagy

As discussed above, since HIV-1 Tat causes an accumulation of autophagosomes in neurons and HIV-1 Nef blocks the fusion of autophagosomes with lysosomes in astrocytes, a drug that targets these proteins could prevent the process of HIV-1-mediated neurodegeneration. Picolinic acid and fusaric acid are two compounds which target and denature HIV-1 Tat by chelating its conserved zinc finger domain leading to inhibition of transactivation [161]. Another Tat inhibitor, didehydro-cortistatin A (dCA), has been shown to reduce residual levels of viral transcription and establish a nearly permanent stage of latency by breaking the Tat-mediated transcriptional feedback loop [162]. Similarly, small molecule antagonists of HIV-1 Nef have been under investigation and might work synergistically with existing cART [163]. An aptamer-bound siRNA against gp120, Tat or Nef may be employed as an efficient drug delivery method to suppress the activity of these proteins [164,165].

## 9. Nano-Formulation of Targeted Drugs

The effectiveness of autophagy-modulating therapy can be increased by the use of different targeted delivery methods including nano-formulations [166]. Designing an effective nano-carrier of desired properties to cross the BBB and be suitable for on-demand cell specific delivery of therapeutic agents is crucial for the development of autophagy-modulating therapy [167,168]. Using magneto-electric nanoparticles (MENP) bound to siBeclin-1, our group showed transmigration of the nano-formulation across an artificial BBB and attenuation of HIV-1 viral titers and inflammation mediated by HIV-1 infection without compromising the physiological integrity of the BBB [169]. Current studies by our group are evaluating additional noninvasive approaches for the delivery of siRNA against Beclin1 to the CNS using animal model [170]. A mixed lineage kinase inhibitor, URMC-099, when used in combination with long lasting nano-formulated cART (Atazanavir), improves the overall outcome of cART [171,172]. URMC-099 facilitates the sequestration and action of long-lasting cART by promoting the nuclear translocation of TFEB. The mechanism of URMC-099 mediated enhancement of cART includes induction of autophagy leading to retention of nanoparticles containing cART within autophagosomes [173]. Although the use of nanotechnology for the delivery of drugs targeting autophagy is largely unexplored, this approach seems to be a promising avenue for the development of HIV-1 therapies and vaccines.

## 10. Conclusions and Perspective

Constant mutation of the HIV-1 genome, capacity of the virus to integrate into the host cellular DNA and establishment of latency are the barriers for eradication or complete cure of HIV-1 patients [174,175]. Furthermore, eradication of HIV-1 reservoirs from the brain has been complicated by toxic effects with higher doses and poor bioavailability with low doses of cART [11,12,13,14]. As such, there is dire need for alternative approaches to the eradication of HIV-1 reservoirs. Currently, several groups of researchers have realized that eradication of HIV-1 reservoirs can only be possible by targeting both cellular pathways and the viral life cycle. One of the potential cellular pathways that can be modulated for HIV-1 therapy and vaccine development is the autophagy pathway [176]. Unfortunately, the complex interaction between HIV-1 and autophagy has made the approach of modulating autophagy for HIV-1 treatment a difficult task [21,147,177,178]. However, studies have shown that modulation and careful monitoring of the autophagy pathway can be implemented as a supplement or alternative to cART in preventing HIV-1 associated neurological disorders [21,169,170,171]. During the initial phase of HIV-1 infection, activation of autophagy could be counterproductive for the host, but beneficial for the virus in many cell types. Therefore, activation of autophagy is pro-viral during the early phase of HIV-1 infection. However, in later phases of infection, HIV-1 inhibition of autophagy promotes the biogenesis of exosomes containing viral products. These exosomes are then beneficial for the virus by priming new cells to be infected and inducing cell death in CD4^+^ T-cells [98,99]. Once HIV-1 latency is established, autophagic induction might complement cART by activating latent viral reservoirs and preventing HIV-1 product export in exosomes [22,78,79,177]. Given the differential interaction of HIV-1 and autophagy, a balanced approach to incorporate autophagy modulation in HIV-1 treatment need to be utilized. As discussed above, autophagy in different CNS cell types is differentially modulated by HIV-1 infection. Therefore, targeting of specific cell types in the brain to modulate autophagy would further increase the potency of this approach.
